# Red blood cell distribution width and peritoneal dialysis-associated peritonitis prognosis

**DOI:** 10.1080/0886022X.2020.1786401

**Published:** 2020-07-02

**Authors:** Peng He, Jin-ping Hu, Huan Li, Xiu-juan Tian, Li-jie He, Shi-ren Sun, Chen Huang

**Affiliations:** a Department of Nephrology, Xijing Hospital, The Fourth Military Medical University, Xi’an, China; b Department of Nephrology, Shaanxi Provincial Secondary People’s Hospital, Xi’an, China

**Keywords:** Red blood cell distribution width, peritoneal dialysis-associated peritonitis, peritoneal dialysis, chronic kidney disease

## Abstract

**Objective:**

Red blood cell distribution width (RDW) is a parameter of the heterogeneity of circulating erythrocyte size. Recent researches have pointed out a link among RDW, chronic kidney disease, and inflammation. We sought to investigate the prognostic value of baseline RDW in patients with peritoneal dialysis-associated peritonitis (PDAP).

**Methods:**

Our study included 337 peritonitis episodes experienced by 202 patients who were undergoing continuous ambulatory peritoneal dialysis (CAPD) at a single center from 2013 to 2018. Episodes were categorized according to the tertiles of baseline RDW levels (T1, <13.2%; T2, 13.2−14.3%; T3, >14.3%). Routine logistic regression and generalized estimating equation (GEE) were used to estimate the association between RDW and treatment failure, which was defined as relapse/recurrent episodes, catheter removal, or death during therapy.

**Results:**

After adjusting for other potential predictors, RDW exhibited an incremental relationship with the risk of treatment failure. The baseline RDW of T3 indicated a 43% and 52% increased venture of treatment failure in logistic and GEE analyses, respectively, compared with T1. As a continuous variable, the fitting curve based on restricted cubic spiline showed that the relationship was nonlinearly but positively correlated. The multivariate model A (combined RDW with baseline age, albumin, serum ferritin, and duration on CAPD) showed an area under the curve of 0.671 (95% confidence interval, 0.5920.749) for the prediction of treatment failure.

**Conclusions:**

A Higher baseline level of RDW was significantly associated with a greater rate of treatment failure among PDAP episodes independent of other potential predictors.

## Introduction

Peritoneal dialysis-associated peritonitis (PDAP), a common and serious complication of peritoneal dialysis (PD), remains a crucial reason for hospitalization, catheter removal, and mortality among PD patients [1,2]. It is well known that PDAP is the direct or major contributing cause of death in more than 15% of patients on PD [3,4]. To be specific, persistent peritonitis, combined with the inflammatory state inherent in PD patients, may result in structural alterations of peritoneum, membrane malfunction, or even permanent peritoneal sclerosis [5,6]. In view of the poor outcomes of peritonitis, early warning and decision-making are needed. Furthermore, existing studies have highlighted the forecasting value of novel biomarkers for adverse outcomes of PDAP [7,8].

Red blood cell distribution width (RDW) is a parameter that reflects the volumetric heterogeneity of erythrocyte in peripheral blood. Elevated RDW is not only associated with enhancive destruction and ineffective production of erythrocyte, but also indicates the iron deficiency anemia, inflammation, and malnutrition status [9,10]. For the past few years, although the underlying mechanism is not well understood, studies have shown that RDW is closely related to the risk of adverse outcomes in multifarious populations, including patients with cardiovascular disease, sepsis, diabetes mellitus, and acute/chronic kidney disease, or even those undergoing hemodialysis (HD) and PD [9,11–20]. The latest multi-center cohort [18], including 14,323 PD patients, suggested that the RDW of ≥16.5% (after adjustment of clinical and laboratory characteristics) had a 40% and 69% higher risk of death in baseline and time-varying analyses compared with the RDW of 14.5–15.5%, respectively. Furthermore, higher baseline RDW (≥16.5%) was associated with a higher risk of first hospitalization (HR, 1.22; 95% confidence interval [CI] 1.14−1.29). Nevertheless, there has been little evidence to verify the strength of association between RDW and unfavorable prognosis of PDAP.

Thus we retrospected the clinical data of peritonitis episodes in our center of Xijing hospital for 5 years. The relationship between baseline RDW and treatment failure was investigated. We speculate that higher baseline RDW is associated with worse outcomes among PDAP patients independent of demographics characteristics, laboratory confounders, and pathogenic spectrum.

## Methods

From 1 July 2013 to 30 June 2018, we recruited patients diagnosed with PDAP in the department of nephrology, Xijing hospital, China. All the participants started continuous ambulatory peritoneal dialysis (CAPD) as renal replacement theraphy. Our research was implemented in conformity to the principles of the Declaration of Helsinki and its amendments. Relevant data was processed anonymously and personal identifiers were completely wiped off.

PDAP was diagnosed according to the latest 2016 ISPD guidelines [21] if 2 of the 3 items below were met: (i) abdominal pain and/or cloudy dialysate fluid; (ii) dialysate white blood cell count (WBC) >100/uL (after a dwell time of at least 2 h), polymorphonuclear >50%; and (iii) positive dialysate fluid culture. When peritonitis was suspected in our medical center, 10 mL dialysate fluid (intraperitoneal retention time >4 h) was retained under strict aseptic operation and routine examination was performed. 5–10 mL dialysate was injected into aerobic and anaerobic bottles for microbiology and antibiotic susceptibility tests. After 3 consecutive specimens were collected, empirical antimicrobial therapy was initiated (cefazolin + ceftazidime). In the meantime, data regarding signs, symptoms and antibiotic dose were recorded daily. If clinical symptoms and signs were not improved within 3 days, the medication regimen would be adjusted according to the test results. Episodes were excluded if they were (i) fungal, (ii) polymicrobial or (iv) mycobacterial peritonitis. We also excluded (v) episodes of insufficient data prior to treatment or follow-up.

Our interested endpoint was treatment failure, defined as catheter removal (including a temporary or permanent switch to hemodialysis), death for any cause, or relapse/recurrence after a peritonitis episode. Relapse was defined as an episode that occurred within 4 weeks of completion of antibiotic therapy with the same organism or one sterile episode, and recurrence was another episode within 4 weeks of completion of therapy with a different organism [21]. Routine blood test was carried out using the automatic hematology analyzer (XN-9000, Sysmex). RDW levels (RDW-CVs) prior treatment were calculated and reported as percentages. The reference range for RDW is 11.5–15.5%. The eligible episodes were categorized by RDW levels (tertiles). Other clinical data were obtained from the medical record system, including the demographic characteristics of the enrolled subjects (age, gender, duration on PD, diabetic status, and residual urine volume), microbiology test, blood rests prior to antibiotic therapy (red blood cell count [RBC], WBC, hemoglobin, albumin, creatinine, potassium, phosphorus, parathormone, and ferritin), and dialysate WBC on day 3 of treatment.

### Statistical analysis

Continuous data were expressed as mean ± standard deviation (SD) or median (interquartile range [IQR]), according to the results of a Kolmogorov–Smirnov test for normality, with categorical data as N (percentages). The significant trends of adverse outcomes were tested by Mantel–Haenszel Chi-square test across the tertiles of baseline RDW. In order to evaluate the covariate-adjusted relationship between RDW levels and treatment failure, binary logistic regression and generalized estimation equation (GEE) analyses were conducted on the data respectively. GEE, a technique used to control for correlations among clustered records, accounted for multiple peritonitis episodes in the same patient. AR (1) was selected as the structure of the working correlation matrix to reflect the interaction between two adjacent episodes. As a continuous variable, the fitting curve based on restricted cubic spline were performed for mapping the associations between baseline RDW levels and the risk of treatment failure events.

Univariate analysis was used for the prelinminary exploration of variables. *p* < .20 suggested a potential predictor for treatment failure. Multivariate analysis was used for model construction and calculating adjusted odds ratio (OR) and 95% confidence interval (CI). The predictive performance of univariate and multivariate models were assessed by receiver operating characteristic curve (ROC) analyses and corresponding accuracy, including area under the curve (AUC), sensitivity, and specificity.

For exploratory research, we conducted stratified analyses according to gender (male or female), infection type (Gram-positive, Gram-negative, or culture negative peritonitis) and binary variables defined by the highest tertile of other clinical parameters (including age, duration on PD, WBC, serum albumin, serum ferritin, and dialysate WBC on day 3) and the combined 2 lower tertiles. We also performed sensitivity analyses based on the adverse outcomes of peritonitis. A 2-sided *p* value <.05 was considered statistically significant. All statistical processing was carried out by SPSS software (version 22.0) and R software (version 3.6.1).

## Results

### Study cohort

There were 365 peritonitis episodes during our study period. 28 episodes were excluded from the analysis for the following reasons: 12 were polymicrobial infections, 10 were fungal infections, and 6 were missing data. Ultimately, 337 peritonitis episodes experienced by 202 patients were included for statistics. Their median (IQR) age was 45 (21) years, and 58.5% were men. The median duration on PD was 16 (25) months. 72 episodes (19.1%) were identified with treatment failure. Among them, 39 episodes were classified as catheter removal. 26 episodes were relapse or recurrence. 7 episodes were death (Supplemental Table S1).

The median of RDW was 13.7% (1.75%). Table 1 present characteristics of the cohort based on tertiles of RDW (T1, <13.2%; T2, 13.2-14.3%; T3, >14.3%). Of all peritonitis episodes, 111, 117, and 109 occurred in T1, T2 and T3. The corresponding episodes of treatment failure were 16, 26 and 30, respectively. The incidence rate of treatment failure was higher in participants with high levels of RDW (Mantel–Haenszel Chi-square, 5.615; *r* = 0.129; *p* for trend = .018). Similar result was calculated when analyzed catheter removal/death (Mantel–Haenszel Chi-square, 6.106; *r* = 0.135; *p* for trend = .013).

**Table 1.** Clinical characteristics of 337 episodes of peritonitis by RDW group.

Characteristics	Overall	RDW		
		T1 (<13.2%)	T2 (13.2–14.3%)	T3(>14.3%)
No. of episodes	337	111	117	109
Age, year	45 (21)	42 (20)	44 (21)	49 (20)
Men, no. (%)	198 (58.8)	70 (63.1)	64 (54.7)	64 (58.7)
Hypertension, no. (%)	309 (91.7)	95 (85.6)	109 (93.2)	105 (96.3)
Diabetes mellitus, no. (%)	35 (10.4)	6 (5.4)	13 (11.1)	16 (14.7)
Coronary artery disease, no. (%)	105 (31.2)	28 (25.2)	30 (25.6)	47 (43.1)
Duration on PD, month	16 (25)	14 (22)	18 (30)	18 (26)
Residual urine volume, ml/24h	300 (900)	400 (900)	300 (950)	200 (550)
Red blood cell, 10^12^/L	3.19 ± 0.72	3.10 ± 0.71	3.30 ± 0.68	3.16 ± 0.76
White blood cell, 10^9^/L	6.04 (3.78)	5.82 (3.47)	6.09 (3.52)	6.15 (5.02)
Platelet, 10^9^/L	204 (114)	213 (50.5)	197 (111)	207 (116)
Hemoglobin, g/L	94.58 ± 20.22	93.35 ± 19.71	98.03 ± 19.37	92.12 ± 21.27
Albumin, g/L	28.28 ± 6.89	28.35 ± 6.29	29.63 ± 7.15	26.75 ± 6.94
Serum creatinin, umol/L	708 (326)	749 (270)	684 (348)	702 (353)
Serum uric acid, umol/L	320 (112)	315 (103)	320.5 (109)	323 (127)
Cholesterol, mmol/L	3.74 (1.34)	3.60 (1.37)	3.92 (1.36)	3.85 (1.32)
Ferritin, ug/L	353 (416)	320.50 (411.75)	365.00 (331.00)	393.00 (487.75)
Potassium, mmol/L	3.78 (1.08)	3.78 (0.85)	3.82 (1.22)	3.60 (1.08)
Phosphorus, mmol/L	1.30 (0.57)	1.23 (0.54)	1.33 (0.51)	1.36 (0.51)
Parathormone, ng/L	204.80 (203.50)	180.45 (152.03)	255.10 (290.75)	193.55 (182.17)
Dialysate white blood cell on day 3, 10^6^/L	153 (436)	178 (623)	162 (466)	109 (365)
Infection type, no. (%)				
Gram-positive peritonitis	202 (59.9)	61 (55.0)	79 (67.5)	62 (56.9)
Gram-negative peritonitis	61 (18.1)	26 (23.4)	15 (12.8)	20 (18.3)
Culture negative peritonitis	74 (22.0)	24 (21.6)	23 (19.7)	27 (24.8)
Treatment failure during episode, no. (%)	72 (19.1)	16 (4.2)	26 (6.9)	30 (8.0)
Catheter removal/death during episode, no. (%)	39 (11.6)	5 (4.5)	18 (15.4)	16 (14.7)
Death, no. (%)	7 (2.1)	2 (1.8)	1 (0.9)	4 (3.7)
Relapse/recurrence during episode, no. (%)	26 (6.9)	9 (2.4)	7 (1.9)	10 (2.7)

RDW: red blood cell distribution width; PD peritoneal dialysis.

### Independent predictors for treatment failure

In routine logistic regression (Table 2, Supplemental Table S2), multivariate analyses indicated that RDW (OR, 1.40; 95% CI, 1.12–1.74; *p* = .003), duration on PD (OR, 1.22; 95% CI, 1.00–1.48; *p* = .048) and dialysate WBC on day 3 (OR, 1.46; 95% CI, 1.17–1.82; *p* = .001) were independent risk factors for treatment failure. In GEE models (Table 2), the predictive value of RDW (OR, 1.44; 95% CI, 1.18–1.76; *p* < .001), duration on PD (OR, 1.23; 95% CI, 1.01–1.51; *p* = .044) and dialysate WBC on day 3 (OR, 1.45; 95% CI, 1.17–1.80; *p* = .001) were also observed.

**Table 2.** Logistic and generalized estimated equation analyses independent risk factors related to treatment failure.

Variables	Logistic regression				Generalized estimated equation			
	Univariate OR (95% CI)	*p* Value	Multivariate OR (95% CI)	*p* Value	Univariate OR (95% CI)	*p* Value	Multivariate OR (95% CI)	*p* Value
RDW, per 1% increase	1.45 (1.20 to 1.75)	<.001	1.40 (1.12 to 1.74)	.003	1.44 (1.21 to 1.72)	<.001	1.44 (1.18 to1.76)	<.001
Age, per 1 year increase	1.02 (1.00 to 1.04)	.117	1.01 (0.98 to 1.03)	.484	1.01 (0.99 to 1.03)	.210	NI	–
Duration on PD, per 1 year increase	1.16 (0.99 to 1.36)	.061	1.22 (1.00 to 1.48)	.048	1.20 (1.02 to 1.42)	.027	1.23 (1.01 to 1.51)	.044
WBC, per 10^9^/L increase	1.05 (0.97 to 1.14)	.217	NI	–	1.07 (0.99 to 1.16)	.092	0.98 (0.89 to 1.08)	.686
Albumin, per 1 g/L increase	0.96 (0.92 to 1.00)	.052	0.97 (0.92 to 1.02)	.173	0.96 (0.93 to 1.00)	.049	0.96 (0.92 to 1.01)	.133
Ferritin, per 10^2^ ng/ml increase	1.05 (1.00 to 1.11)	.049	1.03 (0.97 to 1.10)	.314	1.05 (1.01 to 1.10)	.030	1.04 (0.99 to 1.09)	.191
Dialysate WBC on day 3, per 10^3^/ul increase	1.32 (1.11 to 1.56)	.002	1.46 (1.17 to 1.82)	.001	1.28 (1.08 to 1.53)	.005	1.45 (1.17 to 1.80)	.001
Infection type								
Gram positive	1.00 (referent)	–	1.00 (referent)	–	1.00 (referent)	–	1.00 (referent)	–
Gram negative	2.00 (1.03 to 3.86)	.040	1.06 (0.44 to 2.58)	.897	1.77 (0.89 to 3.54)	.105	0.94 (0.41 to 2.17)	.892
Culture negative	1.65 (0.87 to 3.11)	.124	1.07 (0.48 to 2.40)	.864	1.64 (0.84 to 3.20)	.150	1.03 (0.40 to 2.66)	.946

CI: confidence interval; NI: not included; OR: odds ratio; WBC: white blood cell.

### Association of baseline RDW with treatment failure

**Figure 1.**
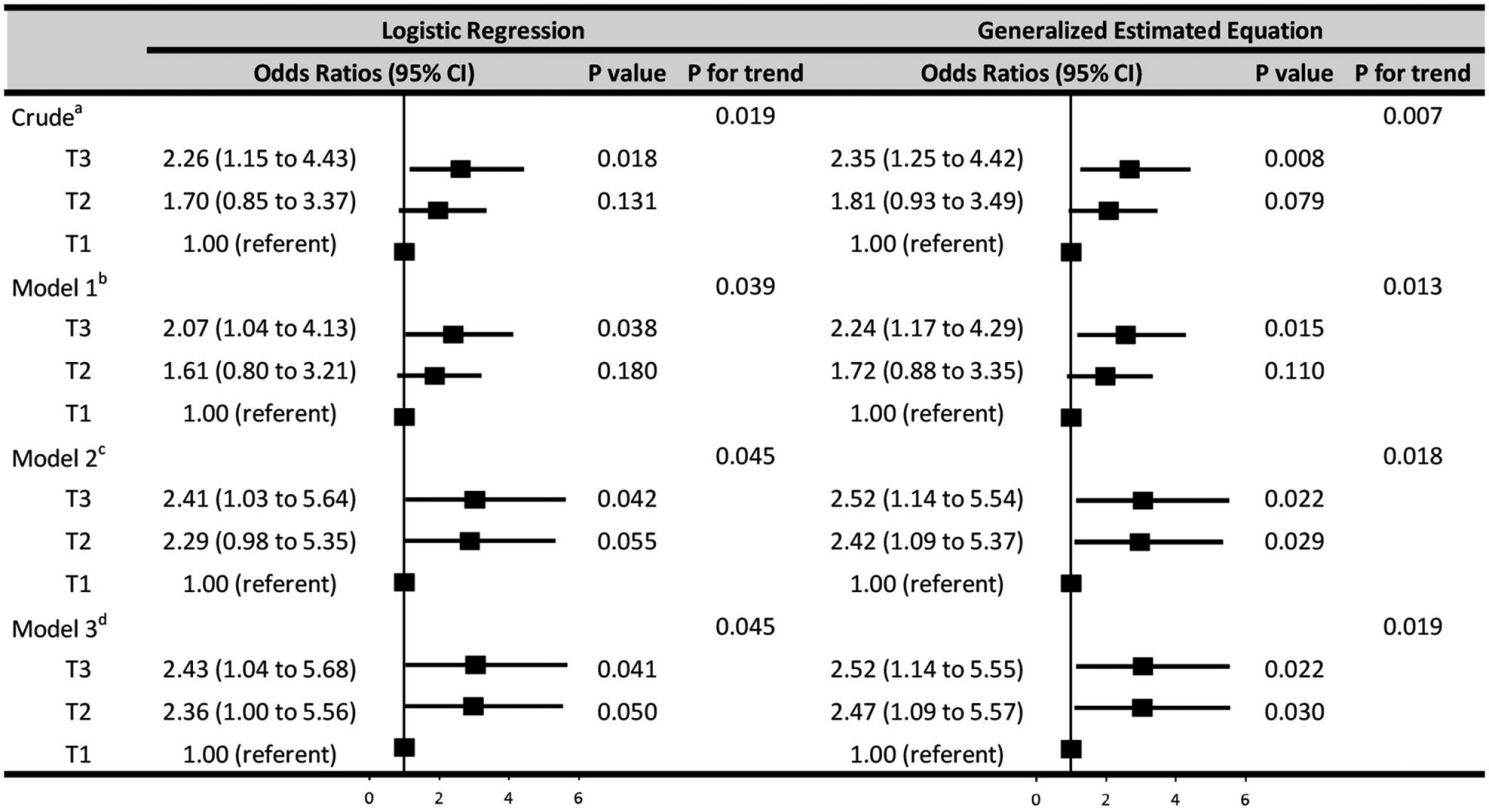
Associations between 3-level categorical variable of baseline RDW and treatment failure. The 3-level categorical variable was defined by tertiles of the baseline red blood cell distribution width (RDW) levels (T1, T2, and T3). The odds ratios were calculated by logistic regression and generalized estimation equation analyses. Crude^a^, no variable was adjusted. Model 1^b^, the odds ratios of RDW were adjusted for demographic characteristics (age, duration on peritoneal dialysis [PD]). Model 2^c^, adjusted for demographic characteristics (age, duration on PD) and laboratory parameters (white blood cell count [WBC], albumin, ferritin, and dialysate WBC on day 3). Model 3^d^, adjusted for demographic characteristics (age, duration on PD), laboratory parameters (WBC, albumin, ferritin, and dialysate WBC on day 3), and infection type (Gram-positive, Gram-negative, or culture negative peritonitis).

After adjustment for demographic characteristics (age, duration on PD), laboratory parameters (WBC, albumin, ferritin, and dialysate WBC on day 3), and infection type (Gram-positive, Gram-negative, or culture negative peritonitis), higher levels of RDW (3-level categorical variable) were independently associated with greater rates of treatment failure (logistic regression, *p* for trend = .045; GEE, *p* for trend = .019) (Figure 1). In logistic regression, relative to T1, the risk of treatment failure in T2 and T3 was higher, that the odds ratios were 2.36 (95% CI, 1.00–5.56; *p* = .050) and 2.43 (95% CI, 1.04–5.68; *p* = .041), respectively. In GEE, the odds ratios of T2 and T3 were 2.47 (95% CI, 1.09–5.57; *p* = .030) and 2.52 (95% CI, 1.14–5.55; *p* = .022), respectively (Figure 1).

**Figure 2.**
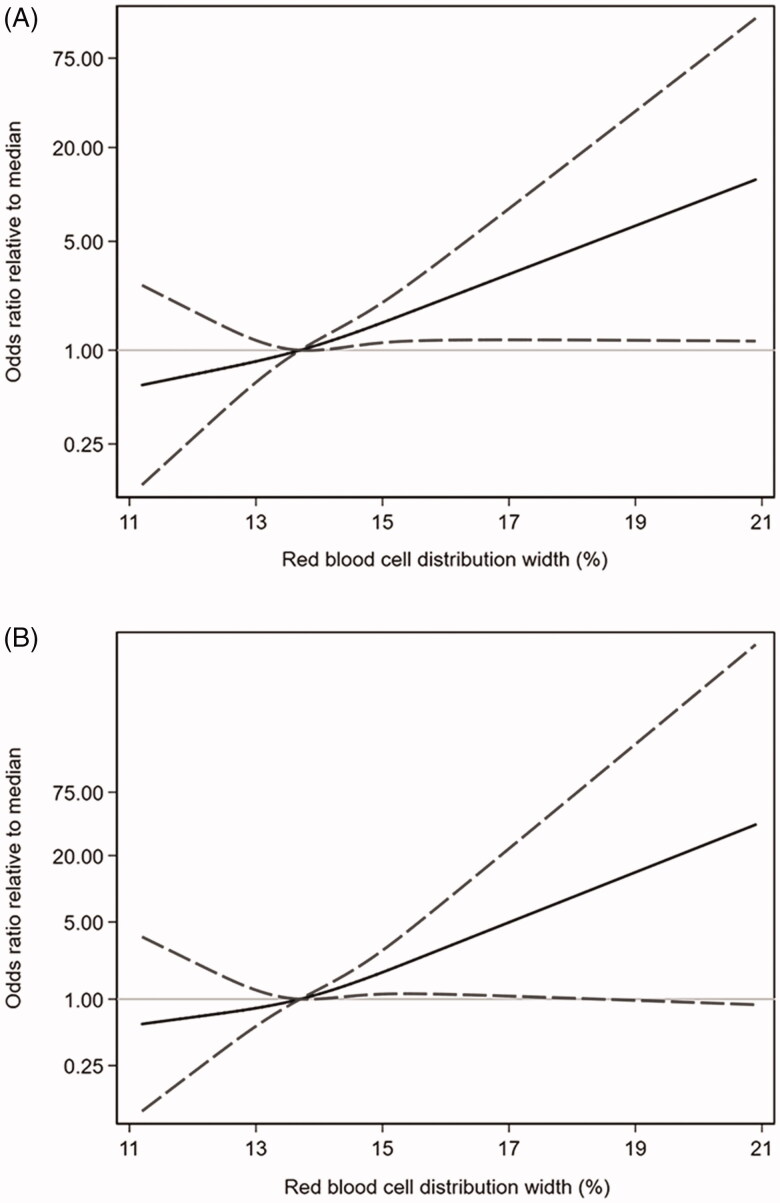
Associations between baseline RDW and treatment failure. Logistic regression (A) and generalized estimation equation (B) models were conducted through restricted cubic splines with knots at the 25th (12.2%), 50th (13.7%), and 75th (15.62%) percentiles. The reference value (the gray lines) was set at the medians. The solid lines indicated the trend of estimated odds ratios, the area between 2 dashed lines indicated the 95% confidence intervals. The odds ratios of baseline red blood cell distribution width (RDW) in 2 models were adjusted for age, duration on peritoneal dialysis, white blood cell count (WBC), albumin, ferritin, dialysate WBC on day 3, and infection type (Gram-positive, Gram-negative, or culture negative peritonitis).

Figure 2 depicted the odds ratios for RDW (continuous variable) related to the median resulting from logistic regression (Figure 2(A)) and GEE (Figure 2(B)) analyses in the whole study population. After adjustment for other potential predictors, RDW turned out to be no linerly but positively associated with the risk of treatment failure events.

### Predictive performence of univariate and multivariate models

As shown in Table 3, the predictive performance of RDW (AUC, 0.618; 95% CI, 0.543–0.693) was slightly lower than dialysate WBC on day 3 (AUC, 0.638; 95% CI, 0.560–0.717), but higher than ferritin (AUC, 0.614; 95% CI, 0.530–0.698) and albumin (AUC, 0.577; 95% CI, 0.501–0.653) in univariate analyses. The optimal cutoff value of RDW was 15.65% with a sensitivity of 0.25 and specificity of 0.94. In multivariate analyses, RDW, dialysate WBC on day 3, and infection type were orderly brought into the initial model by reference to the time sequence of test results. Model A combined RDW and initial model (age, PD duration, albumin, and ferritin) with an AUC of 0.671 (95% CI, 0.592–0.749). The predictive performance was significantly enhanced compared with the initial model (AUC, 0.635; 95% CI, 0.555–0.716) alone. After the development of model B by included dialysate WBC on day 3 in model A, we noticed the predictive value further improved (AUC, 0.719; 95% CI, 0.640–0.799). Nevertheless, the overall predictive accuracy did not dramatically change after the cooptation of infection type (AUC, 0.720; 95% CI, 0.641–0.799) (Delong’s test, Z = −0.538, *p* = .591).

**Table 3.** Predictive value of univariate and multivariate models for treatment failure.

	AUC (95% CI)
Univariate models	
Dialysate WBC on day 3	0.638 (0.560 to 0.717)
RDW (prior treatment)	0.618 (0.543 to 0.693)
Ferritin (prior treatment)	0.614 (0.530 to 0.698)
Albumin (prior treatment)	0.577 (0.501 to 0.653)
Platelet (prior treatment)	0.573 (0.499 to 0.648)
WBC (prior treatment)	0.534 (0.457 to 0.612)
Creatinin (prior treatment)	0.513 (0.431 to 0.595)
Hemoglobin (prior treatment)	0.499 (0.420 to 0.578)
Multivariate models	
Initial Model^a^	0.635 (0.555 to 0.716)
Model A: Initial Model^a^ + RDW	0.671 (0.592 to 0.749)
Model B: Model A + Dialysate WBC on day 3	0.719 (0.640 to 0.799)
Model C: Model B + Infection type	0.720 (0.641 to 0.799)

AUC: area under the receiver operating characteristic curve; CI: confidence interval; RDW: red blood cell distribution width; WBC: white blood cell count.

^a^ Including age, duration on peritoneal dialysis, serum albumin, and serum ferritin.

**Figure 3.**
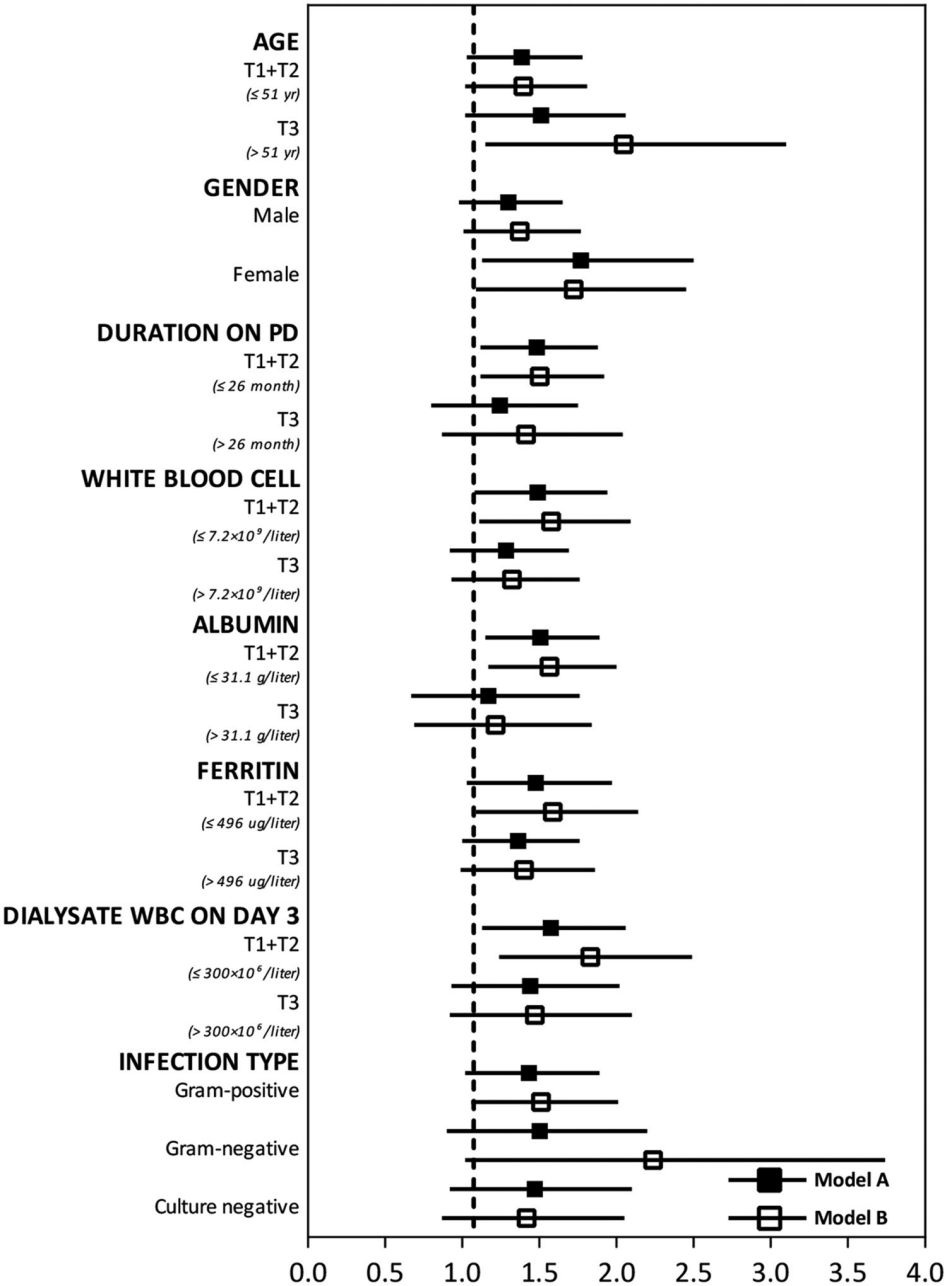
Stratified analyses for the adjusted odds ratios of baseline RDW in model A and model B. The stratified analyses were performed according to gender (male or female), infection type (Gram-positive, Gram-negative, or culture negative peritonitis), and binary variables defined by the highest tertile (T3) of other clinical parameters (age, duration on peritoneal dialysis [PD], white blood cell count [WBC], albumin, ferritin, and dialysate WBC on day 3) and the combined 2 lower tertiles (T1 + T2). The odds ratio of red blood cell distribution width (RDW) in model A was adjusted for age, duration on peritonieal dialysis (PD), albumin, and ferritin. The odds ratios in model B was adjusted for age, duration on PD, albumin, ferritin, and dialysate WBC on day 3.

In stratified analyses (Figure 3), the positive relationship between elevated RDW levels and higher rates of treatment failure was validated in the adjusted models (model A and B). We also conducted sensitivity analyses of model A and B according to different adverse events (Supplemental Table S3). The results indicated that, in model A and B, the risk of catheter removal increased by 63% and 66% for each 1% elevation in baseline RDW levels. The AUC were 0.72 (95% CI, 0.62–0.81) and 0.77 (95% CI, 0.67–0.86), respectively. As for relapse or recurrence episodes, the adjusted odds ratios of RDW were 1.10 (95% CI, 0.77–1.55) and 1.12 (95% CI, 0.79–1.59) in model A and model B. The AUC were 0.61 (95% CI, 0.48–0.74) and 0.62 (95% CI, 0.48–0.76), respectively. Although the result of relape/recurrence was not found to be statistically significant, the point estimates of the odds ratios were in the same direction as integral results.

## Discussion

For all we know, this was the first study to evaluate the association between baseline RDW and treatment failure among PDAP episodes. Two predictive models including RDW and other clinical parameters were developed. Our dominated finding was a robust and positive correlation between baseline RDW and the risk of treatment failure events independent of other indicators. In ROC and sensitivity analyses, the predictive performance of model A and model B were considerable and consistent.

RDW has been used to differentiate the causes of anemia in clinical practice. Moreover, the close relationships between RDW and coronary artery disease, ischemic cerebrovascular disease, peripheral artery disease, hypertensive heart disease, atrial fibrillation and heart failure have been extensively delved and covered [22,23]. In recent studies, RDW has also been implicated in the clinical setting of kidney diseases. A retrospective cohort, recruited 109,675 adult maintenance HD patients in the United States, reported that baseline RDW is a stronger predictor of mortality than traditional laboratory markers of anemia [17]. A meta-analysis, incorporated 9 observational studies with a total of 117,047 subjects, suggested that high levels of baseline RDW probably increase the risk of all-cause mortality in CKD patients [19]. Later, parallel findings were uncovered in PD populations. A single-center retrospective research enrolled 313 incident patients undergoing CAPD from 2006 to 2015 in Taiwan [12]. In Cox regression models, the adjusted hazard ratios (HR) for the high RDW group versus low RDW group were 2.58 (95% CI, 1.31–5.09; *p* = .006) and 3.48 (95% CI, 1.44–8.34; *p* = .006) for all-cause and cardiovascular disease related mortality. The cohort of Soohoo et al. [18] demonstrated that higher levels of baseline and time-varying RDW were associated with increased risk of death and first hospitalization. In our work, after adjusting for other potential risk factors, RDW exhibited an incremental relationship with the risk of treatment failure. The baseline RDW of >14.3% (T3) indicated a 1.43 and 1.52 fold increased venture of treatment failure in logistic and GEE models, respectively, compared with <13.4% (T1). As a continuous variable, the fitting curve showed that the relationship between RDW levels and the risk of treatment failure was nolinearly but positively correlated.

Although the precise pathophysiological mechanisms were not clearly illuminated, several inferences, however, have been suggested to explain how the elevated RDW works as a predictor of adverse events in PDAP. First, persistent inflammation is recognized as a major cause of treatment failure of peritonitis, which can be systematically interpreted by (i) peritonitis itself, especially repeated episodes of Gram negative bacterial peritonitis, (ii) peritoneal inflammation stimulated by biological incompatible factors in dialysate fluid and (iii) uremia related systemic inflammation. Under this circumstances, proinflammatory cytokines make red bone marrow stem cells insensitize to erythropoietin and inhibit erythropoietin to induce erythrocyte maturation and proliferation [24,25]. Existing studies have proved the correlation between elevated RDW and higher level of inflammatory biomarkers both in predialysis and dialysis populations [13,26,27]. Second, hemodynamic overload is one of the common complications of long-term PD. Peritoneal thickening and adhesion, or even enveloping peritoneal sclerosis, caused by sustained peritonitis, may trigger the reduction of peritoneal area and dialysis sufficiency. RDW is also a parameter that reflects morphological plasticity of erythrocyte, which is related to the capacity load through microcirculation [28]. Third, malnutrition is definitely prevalent in patients with end stage renal disease (ESRD), especially for those undergoing long-term dialysis. During peritonitis, extensive proteins are lost due to the typical increase of peritoneal permeability. Interestingly, RDW has been reported to be significantly and inversely correlated with nutritional index in a wide array of medical conditions [29]. Fouth, residual renal function has been widely demonstrated to be beneficial to the survival of PD populations. In the retrospective cohort of Rachel et al. [30], a lower residual renal function (RRF, presented by a greater urinary creatinine clearance) was associated with treatment failure among Gram-positive and culture-negative peritonitis episodes. More importantly, RDW may be a potential indicator of RRF. Yao-Peng et al. found that RRF was greater in the low RDW group than that in the high RDW group in the patients undergoing CAPD [12]. In addition, other mechanisms also have the potential to explain the association between baseline RDW levels and unfavorable outcomes of peritonitis, such as oxidative stress [31], disorder of iron metabolism [32], aging [14,33], thrombosis [28], and so on.

Dialysate WBC has been recommended to be a robust predictor of adverse outcomes related to peritonitis and should be performed again 2–3 days after antibiotic therapy, especially when clinical symptoms do not improved [1,21]. Chow et al. caught sight of using a cut point of dialysate WBC on day 3 (≥1090/mm^3^), the sensitivity was 0.75 and specificity was 0.74, in forecasting treatment failure (defined as catheter loss or peritonitis-related death) [8]. In our work, the predictive value of dialysate WBC on day 3 was higher than baseline RDW in univariate model (AUC, 0.638 vs. 0.618). However, model A (included RDW, age, duration on PD, albumin, and ferritin) showed an AUC of 0.671 (95% CI, 0.592–0.749). In sensitive analyses, it also suggested certain predictive performances for other unfavorable events (AUC for catheter removal, 0.72; for relapse or recurrence, 0.61). Even more important, compared with dialysate WBC on day 3, the advantage of baseline RDW lies in the ease of specimen collection, the simplicity of measurement, and the timeliness of report.

Except for the inherent selection bias in retrospective study, the current work has a few limitations. First, the data were collected from a single medical center with limited case numbers, therefore, center-specific effects cannot be excluded. Because no other matched populations and studies were found, there was no evidence of external validation. Large prospective external validation studies, in different settings, are needed to testify the predictive value of RDW. Second, although the performance and stability of models have been rigorously evaluated, residual confounding or the presence of reporting bias cannot be excluded. Third, the study did not take into account the time-varying RDW (such as the levels on 3 or 5 day after using antibiotics). However, considering the research objective of early prediction for treatment failure events, it is doubtful whether changes in RDW levels in such a short period of time would have a significant impact on the overall performance. And this needs more specific studies to explore.

## Conclusion

In this retrospective cohort, higher baseline levels of RDW were significantly associated with greater risk of treatment failure among PDAP episodes, even after adjusting for other clinical predictors. Given that RDW is an available and convinient hematological parameter, we suggest that it can be used as a valuable classifer for adverse outcomes of PDAP. More large-scale investigations are needed to validate our results and elucidate the underlying mechanisms.

## Supplementary Material

Supplemental Material
