# Supplementary material for: Red blood cell distribution width and peritoneal dialysis-associated peritonitis prognosis
Source: Ren Fail. 2020 Jul 2;42(1):613–21. doi: 10.1080/0886022X.2020.1786401 (PMC7946038; doi:10.1080/0886022X.2020.1786401)
Supplement: Supplemental Material [file IRNF_A_1786401_SM0250.pdf]

Red Blood Cell Distribution Width and Peritonial Dialysis-Associated Peritonitis Prognosis

(Supplemental Tables)

Supplemental Table S1. Clinical characteristics of 337 episodes of peritonitis

Characteristics	Success	Failure	Overall
No. of episodes	265	72	337
Age, yr	44 (21)	48 (20)	45 (21)
Men, no. (%)	154 (58.11)	44 (61.11)	198 (58.8)
Hypertension, no. (%)	244 (92.1)	65 (90.3)	309 (91.7)
Diabetes mellitus, no. (%)	28 (10.57)	7 (9.72)	35 (10.4)
Coronary artery disease, no. (%)	80 (30.2)	25 (34.7)	105 (31.2)
Duration on PD, month	15 (24)	20 (27)	16 (25)
Residual urine volume, ml/24h	300 (850)	200 (1000)	300 (900)
Red blood cell, 10^{12}/liter	3.19 ± 0.72	3.17 ± 0.71	3.19 ± 0.72
White blood cell, 10^{9}/liter	5.98 (3.78)	6.20 (4.20)	6.04 (3.78)
Platelet, 10^{9}/liter	200 (117)	223 (99)	204 (114)
Hemoglobin, g/liter	94.67 ± 20.02	94.22 ± 21.09	94.58 ± 20.22
Albumin, g/liter	28.66 ± 6.92	26.87 ± 6.67	28.28 ± 6.89
Serum creatinin, umol/liter	706 (305)	735.5 (390.5)	708 (326)
Serum uric acid, umol/liter	321.5 (116)	312 (93)	320 (112)
Cholesterol, mmol/liter	3.77 (1.35)	3.65 (1.08)	3.74 (1.34)
Ferritin, ug/liter	329 (364)	479 (468)	353 (416)
Potassium, mmol/liter	3.76 (0.99)	3.84 (1.23)	3.78 (1.08)
Phosphorus, mmol/liter	1.31 (0.60)	1.27 (0.41)	1.30 (0.57)
Calcium, mmol/liter	2.00 (0.23)	1.99 (0.30)	2.00 (0.26)

Parathormone, ng/liter	204.30 (217.80)	213.75 (190.73)	204.80 (203.50)
Dialysate white blood cell on day 3, 10^6/liter	129 (299)	245 (1250)	153 (436)
Infection type, no. (%)			
Gram-positive	167 (63.02)	35 (48.61)	202 (59.9)
Gram-negative	43 (16.23)	18 (25.00)	61 (18.1)
Culture negative	55 (20.75)	19 (26.39)	74 (22.0)

Abbreviations: RDW, red blood cell distribution width; PD, peritoneal dialysis.

Supplemental Table S2. univariate analyses of independent risk factors related to treatment failure

Variables	Routine Logistic Regression			Generalized Estimated Equation		
	Odds Ratios	95% CI	P value	Odds Ratios	95% CI	P value
RDW, per 1% increase	1.45	1.20 to 1.75	**< 0.001**	1.44	1.21 to 1.72	**< 0.001**
Age, per 10 yr increase	1.16	0.96 to 1.41	**0.117**	1.13	0.93 to 1.38	0.210
Gender (female)	0.88	0.52 to 1.50	0.647	0.91	0.52 to 1.59	0.745
Hypertension (yes)	0.80	0.33 to 1.96	0.625	0.72	0.25 to 2.03	0.531
Diabetic status (yes)	0.91	0.38 to 2.18	0.835	0.87	0.36 to 2.12	0.764
Coronary artery disease (yes)	1.23	0.71 to 2.13	0.462	1.14	0.62 to 2.08	0.676
Duration, per 1 yr increase	1.16	0.99 to 1.36	**0.061**	1.20	1.02 to 1.42	**0.027**
Residual urine volume, per 100 ml/24h increase	1.00	0.95 to 1.04	0.836	0.99	0.94 to 1.04	0.637
Red blood cell count, per 10^{12}/liter increase	0.96	0.67 to 1.38	0.820	0.93	0.63 to 1.38	0.716
White blood cell count, per 10^{9}/liter increase	1.05	0.97 to 1.14	0.217	1.07	0.99 to 1.16	**0.092**
Platelet, per 10^{11}/liter increase	1.06	0.87 to 1.28	0.580	1.06	0.91 to 1.25	0.454
Hemoglobin, per 10 g/liter increase	0.99	0.87 to 1.13	0.867	0.98	0.84 to 1.14	0.764
Alubmin, per 10 g/liter increase	0.68	0.46 to 1.00	**0.052**	0.68	0.46 to 1.00	**0.049**
Creatinin, per 100 ummol/liter increase	1.01	0.91 to 1.12	0.885	1.03	0.91 to 1.16	0.659
Uric acid, per 100 ummol/liter increase	1.02	0.77 to 1.35	0.886	1.05	0.81 to 1.36	0.704
Cholesterol, per 1 mmol/liter increase	0.84	0.63 to 1.13	0.257	0.83	0.62 to 1.12	0.220
Potassium, per 1 mmol/liter increase	1.10	0.81 to 1.49	0.553	1.19	0.86 to 1.63	0.299

Phosphorus, per 1 mmol/liter increase	1.10	0.60 to 2.03	0.748	1.20	0.65 to 2.22	0.556
Parathormone, per 100 ng/liter increase	0.97	0.84 to 1.11	0.619	0.97	0.85 to 1.10	0.622
Ferritin, per 100 ug/liter increase	1.05	1.00 to 1.11	**0.049**	1.05	1.01 to 1.10	**0.030**
Dialysate WBC on day 3, per 1000/ul increase	1.32	1.11 to 1.56	**0.002**	1.28	1.08 to 1.53	**0.005**
Infection type						
Gram-positive peritonitis	1.00	referent	—	1.00	referent	—
Gram-negatie peritonitis	2.00	1.03 to 3.86	**0.040**	1.77	0.89 to 3.54	**0.105**
Culture negative peritonitis	1.65	0.87 to 3.11	**0.124**	1.64	0.84 to 3.22	**0.148**

Supplemental Table S3. Sensitivity analyses for the predictive performance of model A and model B

Adverse Events	Patient No. *events/total*	Adjusted ORs[c] (95% CI)	AUC (95% CI)	Sensitivity	Specificity
**Treatment failure**	72/337				
Model A[a]		1.35 (1.10 to 1.67)	0.67 (0.59 to 0.75)	0.46	0.81
Model B[b]		1.40 (1.12 to 1.74)	0.72 (0.64 to 0.80)	0.61	0.75
**Catheter removal/death**	46/337				
Model A[a]		1.51 (1.18 to 1.92)	0.71 (0.62 to 0.80)	0.59	0.76
Model B[b]		1.61 (1.23 to 2.09)	0.78 (0.70 to 0.87)	0.79	0.63
**Catheter removal**	39/330				
Model A[a]		1.63 (1.25 to 2.12)	0.72 (0.62 to 0.81)	0.81	0.54
Model B[b]		1.66 (1.26 to 2.19)	0.77 (0.67 to 0.86)	0.86	0.53
**Relapse/recurrence**	26/291				
Model A[a]		1.10 (0.77 to 1.55)	0.61 (0.48 to 0.74)	0.41	0.81
Model B[b]		1.12 (0.79 to 1.59)	0.62 (0.48 to 0.76)	0.41	0.90

Model A[a], included baseline red blood cell distribution width (RDW), age, duration on peritonieal dialysis (PD), albumin, and ferritin.

Model B[b], included baseline RDW, age, duration on PD, albumin, ferritin, and dialysate white blood cell count on day 3.

C, adjusted odds ratios of baseline RDW in models.

Abrreviations: CI, confidence interval; OR, odds ratioROC, receiver operating characteristic curve.
